# Aural Rehabilitation via Telepractice During COVID-19: A Global Perspective on Evolving Early Intervention Practices

**DOI:** 10.5195/ijt.2021.6362

**Published:** 2021-06-22

**Authors:** Rebecca Claridge, Nicholas Kroll

**Affiliations:** 1 Med-El Worldwide Headquarters, Innsbruck, Austria

**Keywords:** Aural Rehabilitation, Early Intervention, Hearing Impairment, Lesson Kits, Telepractice

## Abstract

**Introduction:**

Pre-pandemic, telepractice was not globally implemented despite its effectiveness. Clinicians reported challenges related to technology, confidence, and inadequate resources.

**Objectives:**

To document global telepractice, identify current obstacles and measure the impact of a possible solution. The timing of this research facilitated tracking telepractice changes during the pandemic.

**Methods:**

Two surveys measured practitioners’ experience and attitude towards telepractice. Survey 1 was completed in February-March 2020. Participants then received two specialized lesson kits to trial if desired. Survey 2 was a follow-up after 4–6 weeks.

**Results:**

Between surveys, the proportion of participants providing telepractice increased from 47.6% to 91.7%. The lesson kits were trialled by 74.3%. Their use had a positive impact on three of the top five factors affecting the delivery of telepractice: parent coaching, clinician experience and accessing resources. *Conclusion:* Telepractice was rapidly adopted globally during the pandemic. The specialized resources were helpful in overcoming some of the barriers to delivery.

The unfolding COVID-19 pandemic had made most in-person visits impossible. People with hearing problems were particularly disadvantaged because compulsory mouth-nose masks have a muting effect on speech and prevent lip reading (Dham et al., 2020; Saile & Gregori, 2020). This factor could be one of the reasons why cochlear implant users are more afraid of the pandemic than the general population (Aschendorff et al., 2020). Inability to access the required services, such as audio processor repair, and the subsequent troubles in communication led to distress in families with children who are deaf or hard of hearing (Ayas et al., 2020). Interruptions to the recommended aural rehabilitation process can slow down speech-language development in young children (Sharma et al., 2020; Tohidast et al., 2020).

Telepractice allows equitable uninterrupted access to aural rehabilitation services with the help of digital devices (McCarthy et al., 2019b). It has been successfully practiced by providers of aural rehabilitation for close to two decades (Flett, 2001), with the lack of qualified specialists in rural areas being the main driving force behind the adoption of telepractice in America and Australia (Behl et al., 2017; Cason, 2009; McCarthy et al., 2012; Olusanya, 2006). Over the years, telepractice has been shown to be as effective as in-person intervention (Behl et al., 2017; Blaiser et al., 2013; Constantinescu et al., 2014; Wales et al., 2017). In the largest study of its kind to date, Behl et al. (2017), used objective measures to demonstrate that early intervention via telepractice yielded the same auditory skills and language development outcomes as in-person therapy (Behl et al., 2017). In addition, families reported high levels of satisfaction with the service (Blaiser et al., 2013; Cason, 2009; Constantinescu, 2012; Olsen et al., 2012).

Despite the growing need for telepractice and the accumulating evidence of its effectiveness, some practitioners and families remain skeptical about telepractice (Cason et al., 2012; Cole et al., 2019; Fairweather et al., 2016). Lack of specialized training, resources, and reimbursement were named as factors preventing the wider adoption of telepractice (Cason et al., 2012; Dorsey & Topol, 2016; Keck & Doarn, 2014; McCarthy et al., 2012). COVID-19 created a unique circumstance wherein telepractice has become a necessity. Most existing studies on telepractice focused on single regions or on multiple services delivered to a mixture of adults and children (American Speech-Language-Hearing Association [ASHA], 2016; Hill & Miller, 2012; Mohan et al., 2017). Recent studies describing the state of telepractice during the pandemic have the same limitations (Fong et al., 2020). Our primary objective was to assess aural rehabilitation specialists’ experience and confidence with telepractice. We hypothesized that provision of appropriate therapy resources could address some of the practitioners’ reservations about telepractice. Therefore, our secondary objectives were to document the most important factors affecting telepractice delivery and to evaluate the impact of ready-made digital resources (the MED-EL Remote Lesson Kits). The timing of this study provided the opportunity to evaluate changes in these parameters during COVID-19 around the globe.

## MATERIALS AND METHODS

### SURVEY DEVELOPMENT

The two surveys were aimed at rehabilitation specialists who worked with families with children using hearing devices. The survey questions were developed by the authors. Survey 1 contained 17 questions and covered demographics (country and occupation), experience in in-person rehabilitation, experience in telepractice, perception of telepractice, and factors affecting telepractice delivery. Survey 2 contained 16 questions and covered experience in telepractice, perception of telepractice, and the feedback on MED-EL Remote Lesson Kits (MED-EL GmbH, 2020a) from the previous 4 weeks. The MED-EL Remote Lesson Kits consisted of ready-to-use lesson plans, descriptions of teaching strategies, detailed activity instructions, tips on parent coaching, and activity resources in PDF format and PowerPoint slide decks. Participants were informed that these kits would be available as free downloads on the MED-EL Professional Blog on the completion of the research. Most questions were multiple-choice, e.g., “How many years of experience do you have delivering telepractice to families with children who are using hearing technology?” with five possible answers: “none”; “less than 1 year”; “1 to 2 years”; “2 to 5 years”; and “more than 5 years”. Answers to some questions were Likert items, e.g., “I feel as confident delivering intervention via telepractice as I do with in-person lessons” with possible answers ranging from “strongly agree” to “strongly disagree” and “not sure.” Several questions had open-ended answers, e.g., “Tell us about how you developed or are developing your skills to deliver telepractice,” but they were not included in this report. All questions and answer categories are listed in Appendix A.

### SURVEY AND REMOTE LESSON KIT DISTRIBUTION

The Ethics Committee of the Medical University of Innsbruck provided an ethical exemption for this study as it included no medical research on human subjects. On 18 February 2020 the invitation to opt into the research was distributed through LinkedIn and to MED-EL Professional Blog subscribers. In a snowball-sample method, respondents were individually emailed a link to Survey 1 on Microsoft Forms and were encouraged to share the survey with others to minimize selection bias; thus, it was not possible to calculate a response rate. A link to Survey 2 was emailed to the respondents of Survey 1, approximately 4 weeks following their completion of Survey 1. This time delay between surveys was intended to allow time for telepractice experience, use of the MED-EL Remote Lesson Kits if desired, and then to collect information on how their telepractice intervention had changed. The data were anonymized before being processed. Participation in the research surveys was voluntary. Participants were informed of their rights and company data protection policies, so participating in and submitting the completed questionnaires were considered sufficient evidence of consent. Early access to MED-EL Remote Lesson Kits 1 and 2 was provided following completion of Survey 1, and Remote Lesson Kits 3 and 4 following completion of Survey 2.

### STATISTICS

Statistical analysis was performed using IBM SPSS Statistics Version 24 (IBM, Armonk, New York). Frequency distributions were calculated for all questions except the open-ended ones. Statistical association between answers to different questions was tested using the Chi-Square Test of Independence. For ease of analysis, the answer categories in several questions were collapsed, where appropriate. The affected questions and the collapsed answer categories are listed in Appendix B. Questions 5 and 7 “How many families with children who are using hearing technology do you provide services to in-person/via telepractice?” in Survey 1 did not differentiate between 0 families and 1 family, so these answer categories were excluded from the analysis.

## RESULTS

### DEMOGRAPHICS

Survey 1 was completed by 273 respondents and 105 of them (38.5%) also completed Survey 2. Survey 2 was completed by 109 respondents. The respondents of Survey 1 came from 41 countries, including 22 developing countries. The largest number of respondents came from English-speaking countries: 38.8% came from the USA, 13.2% from Australia, 6.6% from the UK, and 4.8% from Canada. The respondents of Survey 2 came from 29 countries, with the frequency distribution between the countries largely the same as in Survey 1. In Survey 1, 43.6% of the respondents were speech-language pathologists, 29.3% were teachers of the Deaf, 11.7% were audiologists, 12.8% identified themselves as having a combination of these specializations, and 2.6% selected “other” as an answer. In Survey 2, the distribution of specializations was very similar (0.8-2.6% difference between respective categories).

### EXPERIENCE IN TELEPRACTICE

**Figure 1:**
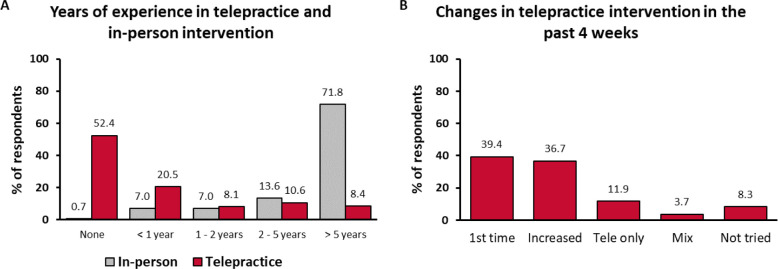
*Note.* Figure 1.A: Years of Experience in Telepractice (Red) and In-person Intervention (Gray), n = 273; Figure 1.B: Changes in telepractice intervention in the past 4 weeks, n = 109; Mix: “I provided a mix of telepractice and face-to-face intervention.”

Respondents’ experience with in-person intervention and telepractice was assessed in Survey 1 in terms of how much experience (in years) they had, how many families they provided services to, and how many sessions per week they conducted. Results showed that initially the respondents had a lot less experience with telepractice than they did with in-person intervention (Figure 1A). Of the respondents in Survey 1, 92.3% had more than 1 year of experience with in-person intervention, with 71.8% having more than 5 years of experience. In contrast, 52.4%, had never delivered telepractice intervention. Of those who had delivered telepractice (n = 130), 43.1% had less than 1 year of experience and 17.7% had more than 5 years of experience. Similar distributions were observed in the countries with the largest number of respondents. A similar pattern was observed regarding the number of families the respondents provided services to and the number of sessions they conducted. In addition, 58.2% of respondents worked with more than 10 families for in-person services and 38.5% had more than one in-person session per day, whereas 8.4% worked with more than 10 families remotely and 9.2% had more than one tele-session per day.

In the time that passed between Survey 1 and Survey 2, the respondents’ exposure to telepractice increased dramatically (Figure 1B). Of those who completed Survey 2, 39.4% tried telepractice for the first time and 36.7% increased their telepractice intervention. Of the respondents, 11.9% delivered telepractice intervention only (i.e., no in-person sessions), 8.3% had not tried telepractice by that time, 27.5% worked with more than 10 families remotely, and 33.9% had more than one tele-session per day.

### CONFIDENCE

**Figure 2:**
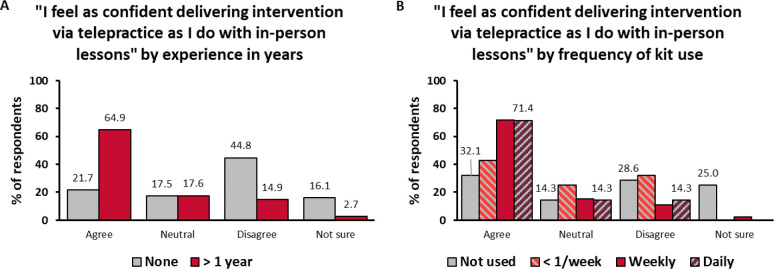
*Note.* Figure 2.A: Confidence with telepractice delivery according to clinician's experience in telepractice (gray: no experience, n = 143; red: more than 1 year of experience, n = 74); Figure 2.B: Confidence with telepractice delivery after 4 weeks according to the frequency of the MED-EL Remote Lesson Kit use, (gray: not used, n = 28; light red, striped: less than once per week, n = 28; red: weekly, n = 46; dark red, striped: daily, n = 7).

Respondents’ confidence with telepractice was assessed in terms of their degree of agreement with the statement “I feel as confident delivering intervention via telepractice as I do with in-person lessons.” We assumed that people felt more confident with in-person intervention than with telepractice, therefore those who agreed with the “as confident” statement must have been more confident with telepractice than those who disagreed. In Survey 1, the respondents were equally likely to agree (35.2%) and to disagree (35.5%) with the statement. Of the respondents, 19.8% selected the answer “neutral” and 9.5% were not sure. Confidence was associated with experience in telepractice (χ^2^: 55.219; df = 6; p < 0.001). The respondents with more than 1 year of experience in telepractice were much more likely to feel confident (64.9%) than the ones without any experience (21.7%) (Figure 2A). The opposite was also true: the inexperienced respondents were more likely to be less confident (44.8%) and unsure (16.1%) than the experienced ones (14.9% and 2.7%, respectively). Confidence level was similarly associated with the number of families in telepractice (χ^2^: 37.465; df = 12; p < 0.001) and with the frequency of tele-sessions (χ^2^: 41.985; df = 12; p < 0.001).

**Figure 3:**
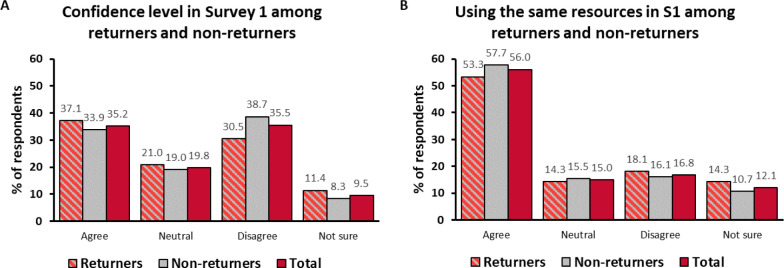
*Note.* Figure 3.A: Confidence with telepractice delivery in Survey 1 among returners (light red, striped, n = 105), non-returners (gray, n = 168) and the total number of respondents (red, n = 273); Figure 3.B: Resource utilization in Survey 1 among returners (light red, striped, n = 105), non-returners (gray, n = 168) and the total number of respondents (red, n = 273).

In Survey 2, the respondents were already more than twice as likely to agree with the statement about confidence than to disagree (54.1% and 21.1%). Of the respondents, 17.4% answered “neutral” and 7.4% were not sure. Of those who increased their telepractice intervention, 77.5% reported feeling confident with it. Even among the first-timers, the largest proportion of respondents (37.2%) already felt as confident with telepractice as with in-person intervention. Confidence was also associated with how often the kits had been used in the previous 4 weeks (χ^2^: 28.363; df = 9; p = 0.001). The respondents who had used the kits daily were a lot more likely to feel confident (71.4%) than those who had not used them at all (32.1%) (Figure 2B). There was no significant association between confidence and the number of families in telepractice (χ^2^: 22.374; df = 15; p = 0.098) or the frequency of tele-sessions in the past 4 weeks (χ^2^: 20.802; df = 12; p = 0.053). There was no apparent sampling bias, because the 105 returning respondents were representative of the total population in Survey 1 and were similar to non-returners in terms of their initial confidence levels (1.9 – 8.2% differences between respective answer categories among returners and non-returners) (Figure 3A).

### OUTCOMES OF TELEPRACTICE

**Figure 4:**
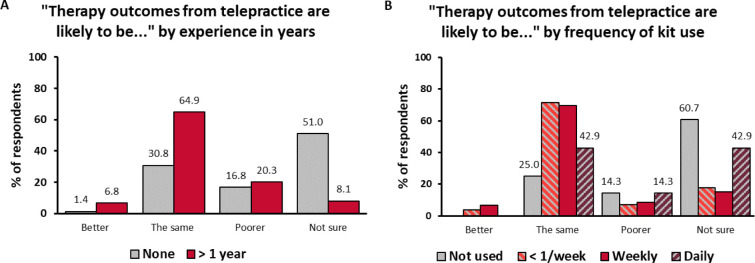
*Note.* Figure 4.A: Telepractice outcome expectations according to clinician's experience in telepractice (gray: no experience, n = 143; red: more than 1 year of experience, n = 74); Figure 4.B: Telepractice outcome expectations according to the frequency of the MED-EL Remote Lesson Kit use, (gray: not used, n = 28; light red, striped: less than once per week, n = 28; red: weekly, n = 46; dark red, striped: daily, n = 7).

In Survey 1, when asked how telepractice outcomes compared to in-person therapy outcomes, 4.4% of respondents expected them to be better, 41.4% to be the same, 20.5% to be poorer, and 33.7% were not sure. Again, the distribution varied substantially depending on the experience (Figure 4A): 30.8% of the respondents with no experience in telepractice expected it to have the same outcomes and 51.0% were not sure, compared to 64.9% of respondents with more than 1 year of experience who expected the outcomes to be the same and 8.1% who were not sure. The association between outcome expectations and experience was significant (χ^2^: 53.030; df = 6; p > 0.001). This variable was also associated with confidence (χ^2^: 59.316; df = 9; p < 0.001). The more confident respondents were more likely to expect the outcomes to be the same (67.7%) and less likely to expect them to be poorer (9.4%) or be unsure (15.6%). The opposite was true for the less confident respondents: 24.7% expected the outcomes to be the same, 32.0% to be poorer, and 42.3% were not sure.

In Survey 2, when asked about the outcomes of telepractice when the MED-EL Remote Lesson Kits were used, the respondents were more likely to expect positive outcomes: 56.9% expected them to be the same, 10.1% to be poorer, and 29.3% were not sure. Respondents who had used the kits any number of times in the previous 4 weeks were more likely to expect the outcomes to be the same than those who had not (Figure 4B). The association between these two variables was significant (χ^2^: 25.040; df = 9; p = 0.003). Outcome expectations were associated with confidence the same way as in Survey 1 (χ^2^: 21.196; df = 9; p = 0.012): the more confident respondents were a lot more likely to expect the outcomes of telepractice with the kits to be the same (71.2%) compared to the less confident ones (43.5%). Again, no apparent sampling bias was observed (3.9 – 5.2% differences between respective answer categories among returners and non-returners).

### USING THE SAME RESOURCES

**Figure 5:**
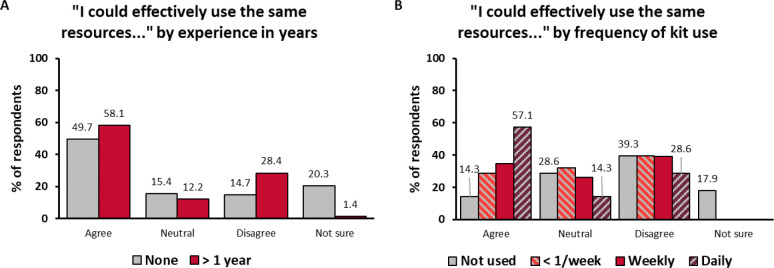
*Note.* Figure 5.A: Resource utilization according to clinician's experience in telepractice (gray: no experience, n = 143; red: more than 1 year of experience, n = 74); Figure 5.B: Resource utilization expectations according to the frequency of the MED-EL Remote Lesson Kit use, (gray: not used, n = 28; light red, striped: less than once per week, n = 28; red: weekly, n = 46; dark red, striped: daily, n = 7).

In Survey 1, the effectiveness of resources used in telepractice was assessed in terms of respondents‘ degree of agreement with the statement “I can (or I think I could) effectively use the same therapy resources for in-person and telepractice sessions.” Of the respondents, 56.0% agreed that they could (or thought they could) use the same resources during both in-person and remote sessions, while 16.9% disagreed. Notably, among the respondents with more than 1 year of experience in telepractice, the proportion of those who agreed was nearly the same (56.0% vs. 58.1%), but the proportion of those who disagreed was larger (16.9% vs. 28.4%) (Figure 5A). The association between resource utilization and experience was significant (χ^2^: 30.123; df = 6; p < 0.001). Resource utilization was also associated with the number of families in telepractice (χ^2^: 22.354; df = 12; p = 0.034) and with the frequency of tele-sessions (χ^2^: 35.333; df = 12; p < 0.001). The respondents who were confident with telepractice were more likely (72.9%) to agree that the same resources could be used; 13.5% of them disagreed. The association between resource utilization and confidence was significant (χ^2^: 57.409; df = 9; p < 0.001).

In Survey 2, the distribution of answers changed: fewer respondents overall agreed that they could use the same resources (29.4%), and more disagreed (38.5%). Of the respondents who used the kits daily, 57.1% agreed that the same resources could be used, compared to 28.6% who disagreed (Figure 5B). The association between resource utilization and how often the kits were used was significant (χ^2^: 19.840; df = 9; p = 0.019). As in Survey 1, the responses on resource utilization were associated with confidence (χ^2^: 27.580; df = 9; p = 0.001), but this time the respondents were much more likely to disagree with the statement or answer “neutral.” A total of 35.6% of the more confident respondents and 60.9% of the less confident respondents disagreed that they could use the same resources. Again, no sampling bias was observed (1.2 – 4.4% differences between respective answer categories among returners and non-returners) (Figure 3B).

### FACTORS AFFECTING TELEPRACTICE DELIVERY

**Figure 6:**
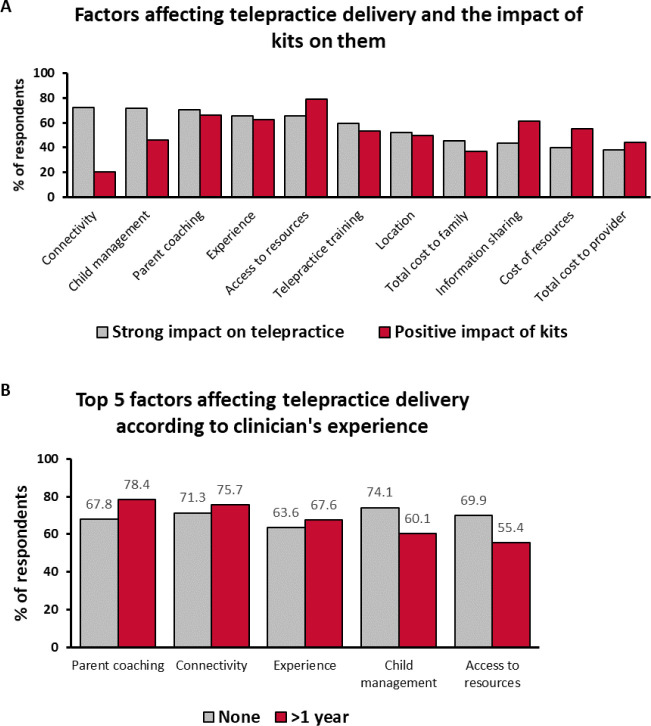
*Note.* Figure 6.A: Proportion of respondents believing that these factors have a strong impact on telepractice delivery (gray, n = 273) and that they were positively affected by the MED-EL Remote Lesson Kits (red, n = 109); Figure 6.B: Top 5 factors having a strong impact on telepractice delivery according to clinician's experience with telepractice (gray: no experience, n = 143; red: more than 1 year of experience, n = 74).

In Survey 1, the respondents were asked to evaluate the impact different factors might have on the telepractice delivery. The factors were ordered according to how many respondents rated them as having a “significant” or “very significant” impact (Figure 6.A). The top 5 factors were: internet connectivity (72.2%), child management (72.0%), clinician use of parent coaching strategies (70.3%), clinician's experience in telepractice (65.6%), and accessing or developing appropriate therapy resources (65.6%). Child management came first among the respondents with no experience in telepractice (74.1%) and only fifth among those with more than 1 year of experience (60.1%) (Figure 6B). The association between this factor and experience was significant (χ^2^: 16.379; df = 8; p = 0.037). In contrast, clinician use of parent coaching strategies was fourth in the no-experience group (67.8%) and first in the experienced group (78.4%), but this association did not reach significance (χ^2^: 12.361; df = 8; p = 0.136). The factor “accessing or developing appropriate therapy resources” was associated with experience (χ^2^: 23.715; df = 8; p = 0.003): it was third in the no-experience group (69.9%) and only sixth in the experienced group (55.4%).

In Survey 2, the respondents were asked to evaluate the impact of the MED-EL Remote Lesson Kits provided on delivery of telepractice. The top 5 factors that the respondents rated as having a “positive” or “very positive” impact were: accessing or developing appropriate therapy resources (78.9%), clinician use of parent coaching strategies (66.1%), clinician's experience in telepractice (62.4%), information sharing (61.5%), and cost of therapy resources (55.0%). Therefore, the kits were most helpful for 3 of the top 5 factors important for telepractice delivery.

## DISCUSSION

We investigated the perception of telepractice among 273 rehabilitation specialists from 41 countries, including 22 developing countries (Survey 1) (United Nations, 2020). Previous studies which focused specifically on remote aural rehabilitation had much smaller samples and were limited to a single center or country (ASHA, 2016; Behl et al., 2017; Blaiser et al., 2013; Fong et al., 2020; McCarthy et al., 2020; Mohan et al., 2017). Survey 1 revealed that more than half of the respondents (52.4%) had not tried telepractice at the start of the COVID-19 pandemic. In the USA, the country with the largest number of respondents, 46.2% had tried telepractice, half of whom had been delivering it for more than 1 year. In comparison, a survey conducted by the American Speech-Language-Hearing Association (ASHA) in 2016 revealed that 63.7% of respondents had experience in telepractice, and almost 82% of them had more than 1 year of experience, but 11.5% provided aural rehabilitation via telepractice (ASHA, 2016).

The COVID-19 pandemic has had a strong impact on the delivery of medical services around the world (Fong et al., 2020; Madden et al., 2020; Saunders & Roughley, 2020; Yellowlees et al., 2020). One of our respondents wrote: “Our practice went from 0% teletherapy to 100% teletherapy in less than a week.” The uniqueness of our study is that it provides a longitudinal analysis of the short-term effects on global telepractice among aural rehabilitation specialists provided with ready-made telepractice resources during the pandemic. Survey 2 revealed a surge in telepractice exposure in a matter of weeks: by May 2020, 39.4% of the returning respondents had tried telepractice for the first time, 36.7% had increased the amount of telepractice intervention they offered, and only 8.3% had not tried telepractice. Of the respondents, 27.5% were now offering telepractice to more than 10 families with children and 33.9% had more than one tele-session per day. Both proportions were below 10% in February 2020. An increase in telepractice due to the pandemic was also reported in Hong Kong: 35% of 135 speech-language pathologists there were providing telepractice at the time of the survey in February-March 2020, and 72.3% of those who had delivered telepractice had started in the previous 3 months (Fong et al., 2020).

Survey 1 identified that confidence with telepractice delivery was associated with experience. The respondents with more than 1 year of experience were three times more likely to feel as confident with telepractice as with in-person intervention. This relationship is supported by the findings of the ASHA survey, which revealed that on average only 1.6% of respondents felt completely unprepared for different aspects of telepractice at the time of the survey, whereas 14.5% felt so when they started delivering telepractice intervention (ASHA, 2016). Although it is normal to feel less confident with new tasks, our second survey revealed that confidence grew in a matter of weeks (35.2% in Survey 1 vs. 54.1% in Survey 2). Of the respondents, 74.3% reported trialing the MED-EL Remote Lesson Kits during this time. Of the rehabilitation specialists who used the kits weekly or daily, 71.7% reported feeling confident with telepractice, compared to 32.1% of those who did not use the kits. The largest proportion of confident respondents was among those who increased their telepractice intervention (77.5%), but even among the first-timers it was quite large (37.2%). This increase is more likely to be attributable to the changes that occurred between the two surveys than to the sampling bias, because the initial level of confidence was similar among returners and non-returners. Both the rising exposure to telepractice and the provision of ready-made resources could have contributed to the increase in confidence, but the design of the study does not allow one to determine the exact contribution of each factor.

The respondents appeared quite skeptical about the outcomes of telepractice despite the accumulating evidence in favor of its effectiveness (Behl et al., 2017; Blaiser et al., 2013; Constantinescu et al., 2014; Wales et al., 2017). Although the largest proportion expected the outcomes to be the same as with in-person intervention (41.4%), 33.7% were not sure and 20.5% expected them to be poorer. Of the American practitioners, 40% were concerned about the quality of early intervention services for children with disabilities if they were delivered via telepractice (Cason et al., 2012). Of speech-language pathologists in Hong Kong, 40.9% indicated “questionable effectiveness” as one of the reasons for not trying telepractice (Fong et al., 2020). In the Hong Kong study, 51.1% of those who tried telepractice thought it was less effective than in-person intervention and 27% agreed that “telepractice is based on current evidence-based practice and is at least equivalent to standard clinical care”. Interestingly, an Australian study revealed that the self-assessed use of family-centered practices did not differ between the practitioners who delivered telepractice and those who delivered in-person intervention. The questionnaire included domains encompassing general and specific information, communication, and interpersonal sensitivity (McCarthy et al., 2020).

Outcome expectations were modified by experience in telepractice following the provision of the MED-EL Remote Lesson Kits. The respondents of Survey 2 who used the Lesson Kits were more positive about the outcomes of telepractice: 66.0% of those who used the kits weekly or daily expected the outcomes to be the same and 9.4% of them expected the outcomes to be poorer. In addition, the respondents with more than 1 year of experience were about twice as likely to expect the outcomes to be the same and over 6 times less likely to be unsure about this question compared to the respondents who had not tried telepractice. Practitioners with higher confidence were much less skeptical and unsure about the outcomes of telepractice: 9.4% of them predicted that the outcomes would be poorer compared to 32% of those who felt less confident. So, the availability of appropriate resources and gaining experience in telepractice could make practitioners more confident of themselves and of the effectiveness of this intervention mode.

In Survey 1, we identified the factors thought to have the greatest impact on telepractice delivery. A total of 11 factors were described as having a significant or very significant impact by 38.1–72.2% of the respondents. Most of the respondents (72.2%) were concerned about connectivity issues. Indeed, this factor is often mentioned as one of the difficulties encountered during tele-sessions (Cole et al., 2019; Fairweather et al., 2016; McCarthy et al., 2019b; Yang et al., 2020). However, Keck and Doarn (2014) demonstrated the feasibility of telepractice using different digital devices across a wide range of speech-language disorders. According to their review, disturbances during real-time videoconferencing had not led to discontinuation of intervention and these could be alleviated using asynchronous technology. It is also important to note that connectivity issues may present significant challenges to telepractice that are difficult for practitioners to directly resolve, but technology for teleconferencing and global connectivity is continually improving. Especially considering shifts to remote working and video conferencing during the pandemic, connectivity challenges may become less of a burden as technology improves and user experience with these platforms increases.

Child attention and behavior management was the second most impactful factor, selected by 72.0% of the respondents. Interestingly, it ranked first among the Survey 1 respondents with no experience in telepractice and only fifth among the respondents with more than 1 year of experience. Although some differences between in-person and tele-sessions are inevitable, interaction strategies can be successfully adapted by the practitioner (Ekberg et al., 2019). Moreover, some parents reported that tablets and other digital devices made their children more engaged during the session (Fairweather et al., 2016).

Approximately 78% of experienced respondents in Survey 1 identified clinician use of parent coaching strategies as the most impactful factor on the delivery of telepractice. The MED-EL Remote Lesson Kits contained guidance on parent coaching and 79% of Survey 2 practitioners who trialed the kits reported that the resources helped them with this important aspect of telepractice. Providing coaching to parents could be key to child management because child-parent interactions come to the forefront in telepractice intervention (Behl et al., 2017; Korfmacher et al., 2008; McCarthy et al., 2019a). Practitioners observe and provide feedback to parents more often during tele-sessions than during in-person visits (Olsen et al., 2012; Stredler-Brown, 2017), which leads to higher parental involvement and engagement (Blaiser et al., 2013; Cole et al., 2019). According to the international consensus statement (Moeller et al., 2013), such family-centered early intervention behaviors are best practice for families who have children with hearing impairment. The results of our study support this idea.

Our hypothesis that provision of specialized therapy resources could be beneficial for practitioners was confirmed. Accessing or developing such resources was the fifth most impactful factor (65.6%), and most respondents (78.9%) reported that the specialized kits provided by MED-EL were very useful. Practitioners with experience in telepractice recognized the need for specialized resources more than those who had not tried telepractice. In a matter of 4 weeks, following the provision of the MED-EL Remote Lesson Kits and after most respondents had introduced tele-sessions to their practice, the overall proportion of respondents who felt they could not use the same resources in telepractice as in in-person therapy had more than doubled. We believe that this increase, like the increase in confidence, can be attributed to the greater exposure to telepractice in combination with the use of the MED-EL Remote Lesson kits that occurred between the two surveys and not to the sampling bias because the initial resource utilization was very similar among returners and non-returners. Interestingly, the practitioners who used the kits more often were more likely to agree that the same resources could be used, which could be an indication that it felt easier to adapt the specialized resources to the familiar in-person setting than the other way round. These kits were adapted from earlier Themed Lesson Kits (MED-EL GmbH, 2020b) that were designed for in-person therapy, so this may also be a reason why the Remote Lesson Kits could be seen as useful for both types of intervention. Our findings are in line with the literature: lack of training, resources, and support was the second most cited reason for not providing telepractice among the practitioners in Hong Kong (Fong et al., 2020). In India, 83.9% of professionals delivering various speech-language therapies believed that the available resources were not sufficient and appropriate for telepractice (Mohan et al., 2017).

According to the results of our surveys, setup and ongoing costs to the family and to the practitioner were least likely to impact telepractice delivery. Fong et al. (2020) also found that only 5.7% of the respondents thought that the costs of telepractice were too high (Fong et al., 2020). Nonetheless, technology expenses and the lack of reimbursement could make telepractice less cost-effective and therefore less attractive (McCarthy et al., 2019b). We did not explicitly ask about this factor in our surveys, but the lack of funding is consistently reported as a barrier to telepractice adoption across literature (Cason et al., 2012; Dorsey & Topol, 2016; Saunders & Roughley, 2020; Smith et al., 2020). Traditionally, telepractice is covered by insurance only in rural areas in countries like the USA and Australia (McCarthy et al., 2012), but even people living in urban centers would benefit from access to remote services, as the COVID-19 pandemic has shown (Smith et al., 2020). Furthermore, telepractice would likely reduce travel burdens and associated costs for patients, which could make aural rehabilitation more easily accessible. However, as with any professional rehabilitation session, telepractice sessions still require significant time and effort from providers. The expected time commitment by practitioners for delivering a telepractice session compared to an in-person session is a topic that has been identified as requiring further investigation (McCarthy et al., 2019b). Preliminary findings suggest that the time commitment is at least equivalent to in-person sessions. Therefore, it would be essential for healthcare systems to provide appropriate funding options for telepractice. Our findings of the rapid increase in telepractice exposure around the world highlight the importance of introducing flexible funding strategies that would allow continuous service delivery, especially for patients for whom timely care is crucial for their development or health (Sharma et al., 2020; Tohidast et al., 2020).

Our study had several limitations. Firstly, the surveys focused in detail on how rehabilitation specialists perceived telepractice, but it is also important to incorporate the family's perspective into the picture. Previous studies consistently reported high levels of satisfaction with telepractice among both practitioners and parents (Cason, 2009; Constantinescu, 2012; McCarthy et al., 2019b). It would be interesting to investigate how attitudes to telepractice changed among patients and whether those who had access to telepractice during the pandemic were satisfied with the service. Secondly, although our surveys were longitudinal, no causality could be inferred from their results. Significant associations between telepractice experience and the practitioner's confidence and outcome expectations could be of either directionality. Controlled experiments could establish the exact nature of these relationships in the future. Lastly, non-native English speakers could have had some difficulties answering questions in English. However, we did not expect it to affect the results because most of the respondents came from English-speaking countries and the questions were formulated in relatively simple English.

## CONCLUSION

COVID-19 has led to a rapid increase in telepractice intervention provided to families with children with impaired hearing. This increase was associated with growing confidence and improved outcome expectations, particularly in practitioners who used the MED-EL Remote Lesson Kits. These ready-made resources containing detailed lesson plans and guidance on parent coaching had a positive impact on some of the most important factors affecting telepractice delivery. These findings may encourage the provision of more telepractice specific resource development, training opportunities and the development of comprehensive reimbursement strategies that would support wider telepractice adoption. Even beyond the scope of the COVID-19 pandemic, the benefits of telepractice could help support more equitable access to effective aural rehabilitation services around the world.

## APPENDIX A

### SURVEY QUESTIONS

**Table A1:** Survey 1 Questions and Answer Categories

n	Question	Answer categories					
1	Country	Open field					
2	I am…	A speech language pathologist	A teacher of the Deaf	An audiologist	Other		
3	How many years’ experience do you have delivering in-person intervention to families with children who are using hearing technology?	None	Less than 1 year	1 to 2 years	2 to 5 years	More than 5 years	
4	How many years’ experience do you have delivering telepractice to families with children who are using hearing technology?	None	Less than 1 year	1 to 2 years	2 to 5 years	More than 5 years	
6	How often in a typical week are you providing in-person intervention to families with children who are using hearing technology?	Less than 1 session per week	1 session per week	2-4 sessions per week	1 session per day	Multiple sessions per day	
7	How many families with children who are using hearing technology do you provide services to via telepractice?	1	2-3	4-6	7-10	More than 10	
8	How often in a typical week are you providing telepractice intervention to families with children who are using hearing technology?	Less than 1 session per week	1 session per week	2-4 sessions per week	1 session per day	Multiple sessions per day	
9	In your practice do families receiving telepractice intervention get seen for therapy more often or less often than families receiving in-person intervention?	My telepractice does not provide both telepractice and in-person services	Telepractice families are seen more often	In-person families are seen more often	Both are offered the same frequency of service	I don’t know	
10	I can (or I think I could) effectively use the same therapy resources for in-person and telepractice sessions.	Strongly agree	Agree	Neutral	Disagree	Strongly disagree	Not sure
11	I have used the MED-EL Lesson kits in telepractice sessions.	Yes	No	I am not familiar with the MED-EL Lesson Kits			
12	To what extent do you think the following impact the delivery of telepractice:						
12a	Set up cost to service provider	Very significant impact	Significant impact	Slight impact	No significant impact	Not sure	
12b	Set up cost to family	Very significant impact	Significant impact	Slight impact	No significant impact	Not sure	
12c	Ongoing cost to service provider	Very significant impact	Significant impact	Slight impact	No significant impact	Not sure	
12d	Ongoing cost to family	Very significant impact	Significant impact	Slight impact	No significant impact	Not sure	
12e	Connectivity (internet) issues	Very significant impact	Significant impact	Slight impact	No significant impact	Not sure	
12f	Accessing or developing appropriate therapy resources	Very significant impact	Significant impact	Slight impact	No significant impact	Not sure	
12g	Cost of therapy resources	Very significant impact	Significant impact	Slight impact	No significant impact	Not sure	
12h	Challenge of information sharing	Very significant impact	Significant impact	Slight impact	No significant impact	Not sure	
12i	Therapy setting, location, environment	Very significant impact	Significant impact	Slight impact	No significant impact	Not sure	
12j	Child attention management	Very significant impact	Significant impact	Slight impact	No significant impact	Not sure	
12k	Child behaviour management	Very significant impact	Significant impact	Slight impact	No significant impact	Not sure	
12l	Clinician completing telepractice training	Very significant impact	Significant impact	Slight impact	No significant impact	Not sure	
12m	Clinician experience in telepractice	Very significant impact	Significant impact	Slight impact	No significant impact	Not sure	
12n	Clinician use of parent coaching strategies	Very significant impact	Significant impact	Slight impact	No significant impact	Not sure	
13	I feel as confident delivering intervention via telepractice as I do with in-person lessons.	Strongly agree	Agree	Neutral	Disagree	Strongly disagree	Not sure
14	Therapy outcomes from telepractice are likely to be:	Poorer than in-person intervention	The same as in-person intervention	Better than in-person intervention	Not sure		
15	Tell us about how you developed or are developing your skills to deliver telepractice.	Open field					
16	How would you describe the process of accessing information to develop your skills in delivering telepractice?	Very easy	Easy	Neutral	Difficult	Very difficult	
17	How can MED-EL help you in delivering telepractice?	Open field					

**Table A2:** Survey 2 Questions and Answer Categories

n	Question	Answer categories							
1	Country	Open field							
2	I am…	A speech language pathologist	A teacher of the Deaf	An audiologist	Other				
3	Over the past 4 weeks, many of us have had to live and work in different ways than we are used to. Which best describes your experience?	I have not had the chance to try telepractice	I commenced telepractice for the first time	I increased my telepractice intervention	I continued to provide telepractice only intervention	I provided a mix of telepractice and face-to-face intervention	Other		
4	Over the past 4 weeks, how many families with children who are using hearing technology have you been providing services to via telepractice?	0	1	2-3	4-6	7-10	More than 10		
5	In the past 4 weeks, how often in a typical week have you been providing telepractice intervention to families with children who are using hearing technology?	Less than 1 session per week	1 session per week	2–4 sessions per week	1 session per day	Multiple sessions per day			
6	Please answer the following question about your recent experience in delivering telepractice to families with children who are using hearing technology. “Over the past 4 weeks I have been able to effectively use the same therapy resources that I would typically use in-person for telepractice sessions.”	Strongly agree	Agree	Neutral	Disagree	Strongly disagree	Not sure		
7	How often did you use the MED-EL Remote Lesson Kits for telepractice?	I did not use them	Less than once a week	Weekly	Daily				
8	Please rate the following elements of the Remote Lesson Kits (very useful, useful, neutral, not so useful, not useful at all, I didn’t look at it).	Learn about the MED-EL remote lesson kits (page 1)	Getting started with remote therapy (page 2)	Key strategies for developing listening skills (pages 3, 4)	Learn about the levels (page 5)	The Lesson Plan (page 6)	The therapist notes for each activity	The printable resources	The slide decks (PPTs)
9	Tell us how you used the Remote Lesson Kits. Tick all that apply.	I didn’t use them	I printed and posted the resources to families	I emailed resources and families printed them	I shared therapist notes with families	I screen-shared the pdfs	I screen-shared the slide decks	Families loaded the slide deck on their device	Other
10	What would have made using the Remote Lesson Kits easier or more practical for you?	Open field							
11	I think that by using the MED-EL Remote Lesson Kits, therapy outcomes from telepractice are likely to be:	Poorer than in-person intervention	The same as in-person intervention	Better than in-person intervention	Not sure				
12	To what extent did using the MED-EL Remote Lesson Kits have an impact on the delivery of telepractice:								
12a	Set up cost to service provider		Very positive impact	Positive impact	No impact	Negative impact	Very negative impact	Not sure	
12b	Set up cost to family	Very positive impact	Positive impact	No impact	Negative impact	Very negative impact	Not sure		
12c	Ongoing cost to service provider	Very positive impact	Positive impact	No impact	Negative impact	Very negative impact	Not sure		
12d	Ongoing cost to family	Very positive impact	Positive impact	No impact	Negative impact	Very negative impact	Not sure		
12e	Connectivity (internet) issues	Very positive impact	Positive impact	No impact	Negative impact	Very negative impact	Not sure		
12f	Accessing or developing appropriate therapy resources	Very positive impact	Positive impact	No impact	Negative impact	Very negative impact	Not sure		
12g	Cost of therapy resources	Very positive impact	Positive impact	No impact	Negative impact	Very negative impact	Not sure		
12h	Challenge of information sharing	Very positive impact	Positive impact	No impact	Negative impact	Very negative impact	Not sure		
12i	Therapy setting, location, environment	Very positive impact	Positive impact	No impact	Negative impact	Very negative impact	Not sure		
12j	Child attention management	Very positive impact	Positive impact	No impact	Negative impact	Very negative impact	Not sure		
12k	Child behaviour management	Very positive impact	Positive impact	No impact	Negative impact	Very negative impact	Not sure		
12l	Clinician completing telepractice training	Very positive impact	Positive impact	No impact	Negative impact	Very negative impact	Not sure		
12m	Clinician experience in telepractice	Very positive impact	Positive impact	No impact	Negative impact	Very negative impact	Not sure		
12n	Clinician use of parent coaching strategies	Very positive impact	Positive impact	No impact	Negative impact	Very negative impact	Not sure		
13	Please describe an activity from the MED-EL Remote Lesson Kits (either Kit 1 or Kit 2) that you found effective.	Open field							
14	The MED-EL Remote Lesson Kits were created from resources originally shared in the free downloadable MED-EL Themed Lesson Kits. Therefore, you could optionally use the resources from the 26 Themed Lesson Kits for telepractice. Did you use them over the past 4 weeks in telepractice sessions?	Yes	No	I am not familiar with the original MED-EL Themed Lesson Kits					
15	How do you feel now about delivering intervention via telepractice? “I feel as confident delivering intervention via telepractice as I do with in-person lessons.”	Strongly agree	Agree	Neutral	Disagree	Strongly disagree	Not sure		
16	What other resources did you use to help you in delivering telepractice?	Open field							

## APPENDIX B

### COLLAPSED ANSWER CATEGORIES

**Table:**

Survey	Question	Original answer categories	Collapsed answer categories
1	3; 4	None Less than 1 year 1 to 2 years 2 to 5 years More than 5 years	None Less than 1 year More than 1 year
	10; 13	Strongly agree Agree Neutral Disagree Strongly disagree Not sure	Agree Neutral Disagree Not sure
	12	Very significant impact Significant impact Slight impact No significant impact Not sure	Positive impact Slight impact No significant impact Not sure
		Child behaviour management Child attention management	Child management
		Setup cost to family Ongoing cost to family	Total cost to family
		Setup cost to service provider Ongoing cost to service provider	Total cost to service provider
	16	Very easy Easy Neutral Difficult Very difficult	Easy Neutral Difficult
2	6; 15	Strongly agree Agree Neutral Disagree Strongly disagree Not sure	Agree Neutral Disagree Not sure
	12	Very positive impact Positive impact No impact Negative impact Very negative impact Not sure	Positive impact No impact Negative impact Not sure
		Child behaviour management Child attention management	Child management
		Setup cost to family Ongoing cost to family	Total cost to family
		Setup cost to service provider Ongoing cost to service provider	Total cost to service provider
